# Advances in Digital Technologies in Dental Medicine: Enhancing Precision in Virtual Articulators

**DOI:** 10.3390/jcm14051495

**Published:** 2025-02-23

**Authors:** Sofia Lobo, Inês Argolinha, Vanessa Machado, João Botelho, João Rua, Junying Li, José João Mendes

**Affiliations:** 1 Egas Moniz Center for Interdisciplinary Research (CiiEM), Egas Moniz School of Health & Science, 2829-511 Almada, Portugal; iargolinha@egasmoniz.edu.pt (I.A.); vmachado@egasmoniz.edu.pt (V.M.); jbotelho@egasmoniz.edu.pt (J.B.); jrua@egasmoniz.edu.pt (J.R.); jmendes@egasmoniz.edu.pt (J.J.M.); 2 Department of Biologic and Materials Sciences & Prosthodontics, University of Michigan School of Dentistry, Ann Arbor, MI 48109, USA; junying@umich.edu

**Keywords:** virtual articulators, intraoral scan, cone beam computed tomography, facial scan

## Abstract

Precision in diagnosis is essential for achieving optimal outcomes in prosthodontics, orthodontics, and orthognathic treatments. Virtual articulators provide a sophisticated digital alternative to conventional methods, integrating intraoral scans, facial scans, and cone beam computed tomography (CBCT) to enhance treatment predictability. This review examines advancements in virtual articulator technology, including digital workflows, virtual facebow transfer, and occlusal analysis, with a focus on Artificial Intelligence (AI)-driven methodologies such as machine learning and artificial neural networks. The clinical implications, particularly in condylar guidance and sagittal condylar inclination, are investigated. By streamlining the acquisition and articulation of digital dental models, virtual articulators minimize material handling errors and optimize workflow efficiency. Advanced imaging techniques enable precise alignment of digital maxillary models within computer-aided design and computer-aided manufacturing systems (CAD/CAM), facilitating accurate occlusal simulations. However, challenges include potential distortions during digital file integration and the necessity for robust algorithms to enhance data superimposition accuracy. The adoption of virtual articulators represents a transformative advancement in digital dentistry, with promising implications for diagnostic precision and treatment outcomes. Nevertheless, further clinical validation is essential to ensure the reliable transfer of maxillary casts and refine digital algorithms. Future developments should prioritize the integration of AI to enhance predictive modeling, positioning virtual articulators as a standard tool in routine dental practice, thereby revolutionizing treatment planning and interdisciplinary collaboration. This review explores advancements in virtual articulators, focusing on their role in enhancing diagnostic precision, occlusal analysis, and treatment predictability. It examines digital workflows, AI-driven methodologies, and clinical applications while addressing challenges in data integration and algorithm optimization.

## 1. Introduction

In recent years, patients have become increasingly aware of their physical appearance and the critical role that maintaining optimal oral health plays in their overall well-being. This heightened awareness is largely due to the pervasive influence of mass media, evolving standards of beauty, and changing social norms that emphasize the importance of esthetics. As a result, there has been a significant increase in the demand for dental care services, not only to restore and improve the functional aspects of the stomatognathic system, but also to enhance facial harmony and smile esthetics [1,2]. The psychological and social implications of dental esthetics are profound, as factors related to dental appearance influence social acceptance, self-image, self-esteem, and overall psychological well-being [1,2].

Therefore, accurate diagnosis is essential for successful esthetic outcomes in prosthodontics, orthodontics, and orthognathic surgery. One of the most effective methods of achieving this predictability is diagnostic waxing, which serves as a fundamental tool for visualizing and simulating potential prosthetic procedures. This technique allows the clinician to provide a comprehensive demonstration of expected treatment results, thereby facilitating informed patient decision-making. It also plays a key role in improving communication and collaboration between the clinician and the dental technician to ensure a more accurate and predictable final prosthetic outcome [1].

With the continued advancement and refinement of digital technologies in dentistry, digital waxing has emerged as a highly effective and practical alternative to traditional manual waxing techniques. This innovative approach significantly reduces the reliance on traditional material handling processes, thereby minimizing the risk of contamination, human error, and inconsistencies in prosthetic design. In addition, digital waxing eliminates certain laboratory production steps that are often prone to inaccuracies due to material distortion, manual dexterity limitations, and environmental factors. By streamlining the workflow, this technology also optimizes time efficiency by reducing the time required for both the waxing procedure itself and the transportation of physical models between the dental clinic and the laboratory, ultimately accelerating the overall treatment timeline and enhancing productivity in clinical practice [1,3].

The adoption of these technologies in prosthodontics presents challenges, including initial investment costs and the learning curve associated with new technology. Despite the benefits, practitioners must evaluate their specific clinical environment, as digital systems require regular updates and can quickly become obsolete. Nonetheless, an all-digital workflow has transformative potential, offering benefits such as reduced production costs, increased efficiency, and improved patient satisfaction within a modernized approach to care [3,4].

In this context, the implementation of a fully digital workflow has the potential to revolutionize the field of fixed prosthodontics by introducing a more precise, standardized, and time-efficient approach to restorative treatment. The adoption of digital methods not only enhances workflow efficiency but also plays a crucial role in minimizing patient discomfort by reducing the number of required clinical visits and improving the accuracy of treatment planning. Furthermore, the incorporation of digital tools provides a more technologically advanced alternative to traditional techniques, aligning with modern demands for high-precision and patient-centered care [3,4,5].

Beyond improving efficiency and accuracy, digital workflows provide significant advantages in diagnostic imaging, treatment predictability, and interdisciplinary coordination. The integration of artificial intelligence (AI) into these workflows further enhances their capabilities, as AI encompasses computational methodologies designed to mimic human cognitive functions, including learning, reasoning, and problem-solving [6,7,8]. By leveraging AI-driven algorithms, digital simulations enable precise preoperative planning, minimizing procedural errors and optimizing clinical decision-making. Moreover, digital dentistry enhances data-driven communication between patients, clinicians, and dental technicians. The integration of digital platforms facilitates seamless collaboration, ensuring prosthetic designs meet both biomechanical and esthetic criteria, ultimately improving treatment outcomes and patient satisfaction [1,2].

This review aims to explore recent advancements in virtual articulator technology and its role in enhancing diagnostic precision and treatment predictability. By analyzing digital workflows, virtual facebow transfer, and AI-driven methodologies, this study seeks to evaluate the impact of virtual articulators in occlusal analysis. Additionally, it addresses the challenges of digital file integration and algorithm optimization while highlighting the potential of virtual articulators to become a standard tool in digital dentistry, supported by AI-driven data processing and predictive modeling.

## 2. Digital Technologies in Dental Medicine

Traditional articulating methods may introduce inaccuracy at each stage of the process, including impression-taking, stone modeling, wax modeling, and casting. In contrast, digital dental systems simplify the process and eliminate the inaccuracy from expansion/distortion of gypsum and occlusal records. In addition, digital impressions are more time-efficient than conventional techniques, easier for less experienced clinicians to use, and generally more comfortable for patients. Intraoral scanning (IOS) has proven to be the preferred method over conventional impressions when used for appropriate indications [9,10,11].

Traditional analog methods are based on a planning process that involves clinical and radiographic assessments, intraoral and extraoral photo evaluations, static and dynamic occlusal analysis, and impressions. However, these conventional analog techniques do not consider the esthetic design of the patient’s smile [12].

A modern approach to implant-supported restorations uses digital impressions to ensure an accurate fit while reducing procedure time compared to traditional techniques that involve multiple manual steps with potential for error. CAD/CAM systems simplify the process and reduce the risk of inaccuracy. Research shows that digital impressions are as accurate as traditional methods, while improving efficiency. They are also more user-friendly for less experienced clinicians, although intraoral scanners can have difficulty capturing details in posterior areas [10,11].

**Figure 1:**
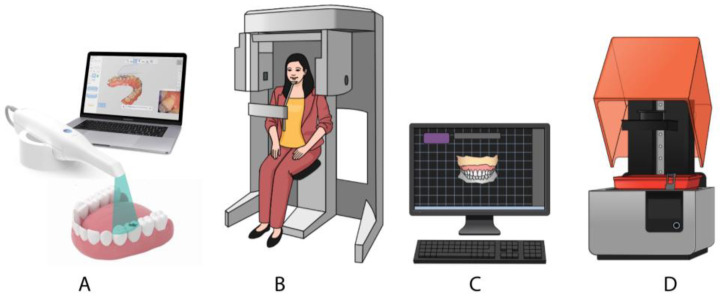
New digital techniques have been introduced in oral rehabilitation. (**A**) intraoral scanner; (**B**) cone-beam computerized tomography; (**C**) 3D software for oral rehabilitation planning and for computer-aided design; (**D**) computer-aided manufacturing as a 3D printer or milling machine.

New digital techniques have been introduced in prosthetics (Figure 1). These include facial scanning (FS), and cone beam computed tomography (CBCT). The development of a virtual three-dimensional (3D) model of a patient requires the seamless integration of data from FS, CBCT, and IOS into a unified and consistent model. FS allows for the integration of a virtual dental setup with a 3D model of the patient’s face, enabling the assessment of how the position, color, and shape of the teeth will affect the facial appearance, with these effects visible on the patient’s face in real time. The potential of digital technologies, such as FS and CBCT, in esthetic treatments is very promising. However, the adoption of these new technologies entails higher costs, and they are still in the early stages of implementation in everyday clinical practice [2,10,11,13,14,15,16].

The occlusal surfaces and overall occlusion of dental restorations play a crucial role in both static and dynamic occlusion. They should be carefully designed to facilitate proper disocclusion during protrusive and lateral excursive movements, ensuring optimal function and comfort for the patient. Using a fully digitized workflow through computer-aided design and computer-aided manufacturing (CAD-CAM) technology, IOS captures highly accurate 3D data of the prepared tooth, adjacent structures, and antagonistic occlusion, facilitating the virtual design of the restoration. Furthermore, crowns fabricated within a digital workflow demonstrate superior marginal adaptation, proximal contacts, occlusal precision, and morphological accuracy [17,18,19,20,21,22].

Virtual facebow transfer and articulator mounting have become possible with the advent of digital technology. These advances are supported by digital data acquisition technologies, including digital photography, facial scanning, CBCT, and jaw tracking systems, which facilitate the precise transfer of the maxillary cast to a virtual articulator for accurate occlusal analysis and simulation [7,23].

In many fields of dentistry, mainly in prosthodontics, orthodontics, and surgery, the assessment of facial soft tissues is critical. Both qualitative and quantitative evaluations of facial esthetics and function are vital for thorough diagnosis and effective treatment planning [24,25].

Not only in the field of prosthodontics have new technologies played an important role, but also in orthognathic surgery and orthodontics. Advances in computing and imaging have enabled the adoption of 3D virtual planning in orthognathic surgery and digital orthodontics, including clear aligner therapy, transforming treatment approaches. These technologies facilitate collaboration between surgeons and orthodontists, allowing for precise pre-procedural planning and simulation. Digital models integrate teeth, soft tissue, and bone, enhancing accuracy in both surgical and orthodontic planning. Additionally, CAD/CAM innovations improve treatment outcomes, while digital tools like Itero Timelapse enable real-time tracking of tooth movement, assisting in treatment adjustments when necessary [26,27].

## 3. Artificial Intelligence in Dental Medicine

### 3.1. Overview of Artificial Intelligence in Dental Medicine

AI research originated with John McCarthy, who introduced the term at the Dartmouth Conference in 1956, symbolizing the origin of the AI scientific field. Subsequent advancements were significant, with researchers focusing on the development of automated reasoning systems, applying AI methodologies to validate mathematical theorems and solve complex algebraic computations [6].

AI encloses computational methodologies designed to mimic human cognitive functions, including learning, reasoning, and problem-solving [6,8,28]. It encompasses the development of systems capable of executing tasks that would typically require human intelligence [29].

### 3.2. Machine Learning and Neural Networks in Prosthodontics

Key subdomains of AI, such as machine learning (ML), deep learning (DL), and artificial neural networks (ANNs), form the foundation of its advanced capabilities [28,30,31].

ML utilizes algorithms to identify statistical patterns and structures within data, enabling predictive analysis on previously unseen datasets. More advanced ML techniques, particularly for data types such as images, incorporate ANNs, which consist of artificial neurons—nonlinear mathematical models designed to process complex information. These neurons are organized into multiple layers, with each layer performing mathematical operations to create a network capable of learning complex features and making sophisticated inferences [28,29,30]. DL refers to neural networks with multiple layers, known as deep neural networks (DNNs). These networks can identify and represent hierarchical features in complex data. For example, in image analysis, they are often used to detect edges, corners, shapes, and larger patterns [29].

### 3.3. Digital Twin Technology: AI-Driven Patient Simulations

Digital Twins in Dental Medicine refer to virtual replicas of physical systems, created using 3D data such as CBCT images, intraoral scans, and digital models. In dentistry, digital twins enable the simulation and analysis of dental and functional structures in a virtual environment, supporting diagnosis, personalized treatment planning, patient education, and even virtual surgical procedures [32,33].

This technology replaces traditional plaster models, which are prone to degradation, loss, and storage challenges. Digital models provide more accurate, accessible, and transferable representations, enhancing clinical workflows. Digital twins also facilitate real-time monitoring, outcome prediction, and dynamic adjustments through the integration of evolving datasets and predictive algorithms. This convergence of physical and digital worlds allows for a deeper understanding of complex dental systems and improved decision-making [31,33].

Although the application of digital twins in dentistry remains in its early stages, it holds significant potential for transforming dental care through personalized, data-driven approaches. Future developments are expected to address current challenges such as standardization of protocols, high implementation costs, and the management of large data volumes, establishing digital twins as a cornerstone of advanced dental practice [31,34].

### 3.4. Future Directions in AI for Dental Medicine

In the field of dentistry, AI-driven software systems have been developed for a wide range of clinical and diagnostic applications, including caries and oral lesion detection, dental plaque identification, periodontal disease diagnosis, automated design of dental prostheses and restorations, and cephalometric analysis [28,30]

The primary goal of AI-based systems is to provide accurate and reliable results for specific tasks, such as detecting pathological conditions or identifying anatomical structures. These advancements have the potential to significantly enhance diagnostic accuracy, treatment efficiency, and workflow optimization in dentistry. However, the ultimate responsibility for diagnosis and treatment remains with dental practitioners, who must rely on their clinical acumen and expertise, whether AI assistance is utilized. The adoption of AI technologies necessitates the establishment of robust regulatory frameworks and guidelines by scientific and professional organizations to ensure their safe, ethical, and effective integration into dental practice [28,29].

Advanced digital data acquisition technologies, including facial scans, intraoral scans, photogrammetry, CBCT, and jaw tracking systems, enable precise and comprehensive capture of patient-specific anatomical structures. The integration of these multimodal datasets facilitates the generation of a 3D virtual patient or a fully detailed digital patient replica. This virtual model serves as a critical resource in advanced clinical workflows, supporting applications such as facial diagnostic waxing, precise implant treatment planning, and the design and fabrication of implant-supported prosthetic restorations [14,25,28].

## 4. Virtual Articulators: Principles and Functions

### 4.1. Comparison Between Virtual and Analog Articulators

The dental articulator is defined as a mechanical device that mimics the temporomandibular joints and jaws, to which upper and lower dental casts can be attached to replicate some or all the movements of the mandible. In the patient’s absence, the articulator can be programmed with patient-specific data, enabling the clinician to design a restoration that precisely fits the individual’s chewing function [35,36].

Articulators, both analog and virtual, remain indispensable instruments in the diagnosis and management of occlusal dysfunction, capable of simulating the jaw movements [37,38].

Articulators have come a long way since their early beginnings in the 18th century when Phillip Pfaff is credited with introducing the first basic model. Today, they have evolved into highly sophisticated tools with adjustable features. With the rise in digital technology and the move toward fully digital workflows in dentistry, mechanical articulators have been gradually replaced or complemented by virtual articulators, reflecting this shift in the dental profession [38].

Shifting from mechanical to virtual articulation eliminates errors associated with material distortion and operator handling during impression-taking, record-making, and cast mounting. When selecting an analog articulator, factors such as sterilization, maintenance, durability, visibility of casts and components, stability during excursions, and balance when opening the upper member must be considered. In contrast, these concerns are eliminated in a digital workflow. The digital environment ensures high precision and accuracy in design and fabrication, with continuous advancements in software and manufacturing expected to further refine these processes [39].

Nevertheless, articulators exhibit inherent limitations, including the approximation of the hinge axis position and the oversimplification of mandibular movements to a unidimensional rotational arc of closure and linear excursive pathways. Despite these limitations, articulators are integral to clinical workflows, providing reliable insights and facilitating efficient treatment protocols [37].

### 4.2. Functionality and Mechanisms of Virtual Articulators

Virtual articulators have been introduced to design restorations with dynamic adjustments and should be considered as an additional diagnostic and treatment plan [23,40,41,42].

Virtual reality, rooted in advanced electronic and computational technologies, has become an integral component of modern dental practice. The virtual articulator (VA) exemplifies the application of this technology in restorative and prosthetic dentistry, significantly mitigating the limitations of conventional physical articulators [35]. The main indications for virtual articulators are personalized diagnostics and the avoidance of typical problems associated with conventional articulators, such as the formation of new occlusal contacts, deformation of materials, errors in the alignment and positioning of dental casts, and challenges in accurately reproducing patient data in 3D [40,41].

The fundamental components common to all VAs involve the acquisition and transfer of the dental arches into the virtual environment, followed by the articulation of the digital models. This process includes capturing a digital impression of the dental arches, recording the static occlusal relationship and dynamic mandibular movements during excursive paths, transferring the maxillary position in relation to the craniofacial structure, and finally mounting the digital models onto the virtual articulator for articulation [41].

The first software to integrate VA was DentCam (Kavo; Hamburg, Germany). It included a device designed to capture mandibular movements and reproduce them as an animation. The incorporation of patient-specific data into the DentCam software (Kavo; Hamburg, Germany) allowed clinicians to visualize and assess both static and dynamic occlusal contacts during mandibular kinematics, enabling detailed analysis of occlusal behavior throughout functional movements [41].

By employing sophisticated data acquisition and modeling techniques, virtual articulators enable precise analysis of static and dynamic occlusal relationships, as well as detailed evaluation of the patient’s maxillomandibular relationship. These software systems utilize virtual reality technology to optimize diagnostic precision and improve therapeutic outcomes in contemporary dental workflows [35].

### 4.3. Technological Software and Platforms

These virtual articulators are present in most dental CAD software, such as Exocad (exocad GmbH, Darmstadt, Germany), 3Shape (3Shape, Copenhagen, Denmark), CEREC (Dentsply Sirona, Charlotte, NC, USA), and Zirkonzahn (Zirkonzahn Worldwide, Gais, Italy) which operate on the same principles as traditional mechanical articulators [42].

#### Programming a Virtual Articulator

It is common practice to position digital models on virtual articulators that are pre-configured with normative data and standardized articulator parameters, which can lead to design errors [17].

There are two possibilities for using the virtual articulator: to set up the models with the program’s default and average values (mathematically simulated) or in a completely adjustable way for each individual [35,41,43,44].

Once programmed for each individual, virtual articulators can accurately reproduce mandibular trajectories [41,43,44]. Therefore, the main disadvantage of using the virtual articulator with the average is that it does not simulate individualized mandibular movements accurately enough. However, it may be more user-friendly and less expensive [41].

Several techniques have been proposed for programming a virtual articulator [42,45,46,47]. It is possible to use a virtual facebow to align the 3D FS with the IOS in a CAD/CAM system and position the digital maxillary model in a virtual articulator to match the facial landmarks [42,47,48]. CBCT can also be used to determine the condylar guidance angles and assist in a virtual articulator in a CAD/CAD system [49,50,51]. Condylar guidance refers to the movement of the mandible guided by the condyle and articular disk as they move along the articular eminence [52], and sagittal condylar inclination is a crucial parameter in oral rehabilitation, defined as the angle formed between the protrusive condylar path and the Frankfort plane [42,53,54,55,56,57]. Thus, the combination of digital data on condylar movements with individual reference planes can help optimize the functional adaptation of prosthetic components [53].

In this regard, software capable of recording protrusive and laterotrusive movements guided by teeth could provide data to design the morphology of occlusal surfaces in restorations, thereby eliminating occlusal interference and ensuring occlusal surfaces that are in harmony with the masticatory muscles [17,58]. There are programs and devices (optical jaw tracking systems) that can record mandibular movements, such as TRIOS (3Shape, Copenhagen, Denmark) and Modjaw^®^ (MODJAW, Villeurbanne, France) [53,59]. The TRIOS 3 and TRIOS 4 (3Shape, Copenhagen, Denmark) IOS have the patient-specific motion (PSM) feature, which captures dynamic occlusion in addition to 3D bite data [59,60]. The Modjaw^®^ (MODJAW, Villeurbanne, France) captures the movements of the patient’s hinge axis during functional mandibular actions and transfers these data, along with the patient-specific reference plane, to the CAD/CAM software [7,28,53].

## 5. Challenges and Limitations

Recent studies indicate that research on the accuracy and reproducibility of maxillary cast transfers using FS and CBCT remains insufficiently explored. While these digital acquisition techniques provide high-resolution 3D data of craniofacial structures, their ability to precisely replicate maxillary spatial positioning within a virtual articulator environment has not yet been fully validated. Challenges such as data resolution variability, segmentation errors, and inconsistencies in landmark identification can lead to registration inaccuracies, which may compromise occlusal analysis and treatment predictability. The accuracy of virtual maxillary cast transfers is crucial for maintaining correct occlusal vertical dimension, intermaxillary relationships, and functional articulation, necessitating further controlled clinical to assess and optimize these methodologies [7,61].

In addition, the alignment of a patient’s digital files using CAD software can introduce distortions in the digital process. A critical challenge in digital prosthodontics and occlusion analysis is the integration and superimposition of multiple digital datasets from IOS, FS systems, and CBCT imaging within CAD software. The alignment of these datasets depends on the precision of surface-matching algorithms, which must accurately synchronize anatomical landmarks while minimizing distortions due to interpolation, or image segmentation errors [28,61,62,63,64]. Even minor deviations in landmark registration can result in discrepancies in occlusal positioning, condylar guidance simulation errors, and inaccuracies in the articulation of prosthetic components. These limitations highlight the need for advanced computational models, machine learning-based error correction, and enhanced AI-driven data fusion techniques to refine the accuracy and stability of digital maxillary cast positioning. It is, therefore, essential that future research prioritize the development of standardized calibration protocols, improved algorithmic precision for automated data superimposition, and enhanced deep learning frameworks. The ultimate goal is to integrate different patient data seamlessly, allowing virtual articulators to accurately replicate mandibular movements and occlusal function. Overcoming these challenges will help digital prosthodontics and occlusion diagnostics become more precise, efficient, and clinically reliable, improving treatment accuracy and long-term prosthetic success [28,61,62,63].

## 6. Future Directions in Virtual Articulator Technology

### 6.1. Virtual Articulators for Multidisciplinary Integration

The digital implementation of virtual articulators has great potential and should be further developed to enhance its use in multidisciplinary cases. These cases often require the collection of large amounts of information from multiple sources, such as dental arches, occlusion, and soft and hard tissues, and the integration of all these data into a unified planning model [33]. The ability to simulate mandibular movements with high precision is crucial for ensuring accurate diagnoses and treatment planning, particularly in complex cases involving prosthodontics, orthodontics, and maxillofacial surgery [35].

Advancements in computational modeling and AI have the potential to further refine the accuracy and predictive capabilities of virtual articulators. By incorporating machine learning algorithms, these systems could analyze patient-specific data to provide real-time adaptive simulations, improving diagnostic accuracy and treatment outcomes. Additionally, cloud-based storage and interoperability with other digital tools, such as CBCT and IOS, would enable seamless data integration across disciplines [41,65].

Virtual articulators have the potential to become a regular part of everyday clinical practice in the future. However, additional clinical studies are required to confirm their precision and their clinical application. Continued validation of these technologies through evidence-based research is essential to establish standardized protocols and regulatory approval for widespread clinical adoption [41,55].

### 6.2. Digital Dentistry in Sustainable Clinical Practices

Sustainability in dental medicine has gained increasing attention due to the environmental impact of traditional clinical and laboratory practices, which often involve high levels of material waste, energy consumption, and transportation-related carbon emissions [56]. The shift toward digital workflows in modern dental practice has emerged as a transformative approach to addressing these sustainability challenges. By integrating advanced digital technologies, such as computer-aided design and computer-aided manufacturing (CAD/CAM), intraoral scanning, and 3D printing, dental professionals can significantly reduce reliance on disposable materials, optimize resource utilization, and decrease the ecological footprint associated with manufacturing and transport processes. These innovations not only enhance clinical efficiency but also contribute to more environmentally responsible practices within the field of dentistry [3,66].

One of the most notable environmental concerns in traditional dentistry is the excessive waste generated by conventional impression-taking techniques. Traditional methods rely on disposable materials such as alginates, silicones, and gypsum-based stone models, which require frequent replacement and disposal after a single use. These materials contribute to clinical waste accumulation, as they are non-biodegradable and cannot be easily recycled. Furthermore, the processing of stone models requires large amounts of water and generates dust and debris, further exacerbating environmental concerns [3,67,68].

Intraoral scanning has emerged as a sustainable alternative, as it eliminates the need for physical impressions by creating a precise, digital representation of the patient’s dentition [68]. This transition not only reduces material consumption but also enhances accuracy and patient comfort while minimizing procedural errors that may lead to retakes and additional waste production [3,66,68].

Additionally, CAD/CAM technology and 3D printing have introduced highly efficient and sustainable manufacturing processes for prosthetic and restorative dentistry. Traditional fabrication methods involve multiple manual steps, including casting, waxing, and layering, which can result in substantial material wastage due to trimming, remakes, and adjustments. Digital manufacturing, on the other hand, optimizes material usage by producing highly precise restorations with minimal excess, ensuring that only the necessary amount of material is utilized. Additive manufacturing techniques, such as 3D printing, further contribute to sustainability, as they create structures layer by layer, reducing the amount of discarded material compared to subtractive milling methods [3,66,69].

The environmental benefits of digital workflows extend beyond material conservation to include energy efficiency and reduction of carbon emissions. Traditional dental workflows require frequent transportation of physical impressions, models, and prosthetic components between clinics and laboratories, contributing to fuel consumption and greenhouse gas emissions [70]. By replacing these physical transfers with digital transmission of intraoral scans and CAD files, clinics can significantly lower their carbon footprint while streamlining communication with laboratories. This not only enhances the speed and efficiency of the fabrication process but also reduces logistical costs and delays associated with transportation [66,70,71].

As the dental industry continues to embrace technological advancements, the role of sustainability in digital dentistry will become increasingly significant. The adoption of eco-friendly materials, energy-efficient machinery, and improved recycling programs for electronic waste will be essential for maximizing the environmental benefits of digital workflows. By integrating these solutions into daily clinical practice, digital dentistry represents a promising pathway toward a more sustainable, efficient, and environmentally responsible approach to patient care [66,70].

## 7. Conclusions

The integration of virtual articulators into digital workflows has significant clinical implications in prosthodontics, orthodontics, and orthognathic treatments. By enabling more accurate transfer of maxillary casts and occlusal relationships, VAs increase diagnostic accuracy and treatment predictability, improving both functional and esthetic outcomes.

From a clinical perspective, the use of digital techniques such as intraoral scanning, facial scanning, and cone beam computed tomography minimizes the errors associated with conventional methods and reduces the risk of discrepancies in prosthetic fabrication and occlusal adjustments. In addition, VAs facilitate better communication between clinicians, technicians, and patients by providing a comprehensive virtual representation of the treatment plan.

However, challenges remain regarding the accuracy of digital maxillary cast transfers, standardization of data integration methods, and validation of condylar motion simulations. Further research is needed to refine these processes and ensure that VA can be reliably incorporated into daily clinical practice. As digital dentistry continues to advance, VAs have the potential to become a standard tool to optimize treatment workflows and improve patient care. Continued clinical validation is essential to fully establish their accuracy, reproducibility, and practical benefits in everyday practice.

## Abbreviations

The following abbreviations are used in this manuscript:

3D	Three-dimensional
AI	Artificial Intelligence
ANNs	Artificial Neural Networks
CAD-CAM	Computer-aided Design and Computer-aided Manufacturing
CBCT	Cone Beam Computed Tomography
DL	Deep Learning
DNNs	Deep Neural Networks
FS	Facial Scan
IOS	Intraoral Scan
ML	Machine Learning
VA	Virtual Articulator

## References

[1] Abduo J., Bennamoun M., Tennant M., McGeachie J. (2016). Impact of Digital Prosthodontic Planning on Dental Esthetics: Biometric Analysis of Esthetic Parameters. J. Prosthet. Dent..

[2] Perroti G., Goker F., Rossi O., Nowakowska J., Russillo A., Beltramini G., Tartaglia G.M., Testiru T., Del Fabbro M., Mortellaro C. (2023). 3D Computed Tomography vs. 2D Radiography: Comparison of 3D Direct Anthropometry with 2D Norm Calculations and Analysis of Differences in Soft Tissue Measurements. Eur. Rev. Med. Pharmacol. Sci..

[3] Chochlidakis K.M., Papaspyridakos P., Geminiani A., Chen C.-J., Feng I.J., Ercoli C. (2016). Digital versus Conventional Impressions for Fixed Prosthodontics: A Systematic Review and Meta-Analysis. J. Prosthet. Dent..

[4] Joda T., Zarone F., Ferrari M. (2017). The Complete Digital Workflow in Fixed Prosthodontics: A Systematic Review. BMC Oral. Health.

[5] Joda T., Bragger U., Gallucci G. (2015). Systematic Literature Review of Digital Three-Dimensional Superimposition Techniques to Create Virtual Dental Patients. Int. J. Oral Maxillofac. Implant..

[6] Xu Y., Liu X., Cao X., Huang C., Liu E., Qian S., Liu X., Wu Y., Dong F., Qiu C.-W. (2021). Artificial Intelligence: A Powerful Paradigm for Scientific Research. Innovation.

[7] Revilla-León M., Zeitler J.M., Kois J.C. (2024). An Overview of the Different Digital Facebow Methods for Transferring the Maxillary Cast into the Virtual Articulator. J. Esthet. Restor. Dent..

[8] Revilla-León M., Gómez-Polo M., Vyas S., Barmak A.B., Özcan M., Att W., Krishnamurthy V.R. (2022). Artificial Intelligence Applications in Restorative Dentistry: A Systematic Review. J. Prosthet. Dent..

[9] Schlenz M.A., Klaus K., Schmidt A., Wöstmann B., Mersmann M., Ruf S., Bock N.C. (2022). The Transfer Accuracy of Digital and Conventional Full-Arch Impressions Influenced by Fixed Orthodontic Appliances: A Reference Aid–Based in Vitro Study. Clin. Oral Investig..

[10] Ahlholm P., Sipilä K., Vallittu P., Jakonen M., Kotiranta U. (2018). Digital Versus Conventional Impressions in Fixed Prosthodontics: A Review. J. Prosthodont..

[11] Burzynski J.A., Firestone A.R., Beck F.M., Fields H.W., Deguchi T. (2018). Comparison of Digital Intraoral Scanners and Alginate Impressions: Time and Patient Satisfaction. Am. J. Orthod. Dentofac. Orthop..

[12] Cattoni F., Mastrangelo F., Gherlone E.F., Gastaldi G. (2016). A New Total Digital Smile Planning Technique (3D-DSP) to Fabricate CAD-CAM Mockups for Esthetic Crowns and Veneers. Int. J. Dent..

[13] Mai H.Y., Choi Y.-D., Lee D.-H. (2019). Digital Approach Integrating 3D Facial Scan and a Virtual Mockup for Esthetic Restorative Treatment: A Case Report. J. Korean Acad. Prosthodont..

[14] Mangano C., Luongo F., Migliario M., Mortellaro C., Mangano F.G. (2018). Combining Intraoral Scans, Cone Beam Computed Tomography and Face Scans: The Virtual Patient. J. Craniofacial Surg..

[15] Saad S.A., Shalaby Y.A., Azer A.S. (2024). Reliability of the Digital Functionally Generated Path Technique for Assessing Occlusal Interferences and Adjusting CAD-CAM Zirconia Crowns: An in Vivo Study. BMC Oral. Health.

[16] Inoue N., Scialabba R., Lee J.D., Lee S.J. (2024). A Comparison of Virtually Mounted Dental Casts from Traditional Facebow Records, Average Values, and 3D Facial Scans. J. Prosthet. Dent..

[17] Li L., Chen H., Li W., Wang Y., Sun Y. (2023). Design of Wear Facets of Mandibular First Molar Crowns by Using Patient-Specific Motion with an Intraoral Scanner: A Clinical Study. J. Prosthet. Dent..

[18] Berrendero S., Salido M.P., Ferreiroa A., Valverde A., Pradíes G. (2019). Comparative Study of All-Ceramic Crowns Obtained from Conventional and Digital Impressions: Clinical Findings. Clin. Oral. Invest..

[19] Carrilho Baltazar Vaz I.M., Pimentel Coelho Lino Carracho J.F. (2020). Marginal Fit of Zirconia Copings Fabricated after Conventional Impression Making and Digital Scanning: An in Vitro Study. J. Prosthet. Dent..

[20] Tabesh M., Nejatidanesh F., Savabi G., Davoudi A., Savabi O., Mirmohammadi H. (2021). Marginal Adaptation of Zirconia Complete-Coverage Fixed Dental Restorations Made from Digital Scans or Conventional Impressions: A Systematic Review and Meta-Analysis. J. Prosthet. Dent..

[21] Yang S., Wu L., Alabkaa B., Lepidi L., Yue L., Li J. (2024). Intraoral Scanner-based Virtual Facebow Transferring: A Chairside Dental Technique. J. Prosthodont..

[22] Úry E., Fornai C., Weber G.W. (2020). Accuracy of Transferring Analog Dental Casts to a Virtual Articulator. J. Prosthet. Dent..

[23] Park J.H., Lee G.-H., Moon D.-N., Kim J.-C., Park M., Lee K.-M. (2021). A Digital Approach to the Evaluation of Mandibular Position by Using a Virtual Articulator. J. Prosthet. Dent..

[24] Cascos R., Ortiz Del Amo L., Álvarez-Guzmán F., Antonaya-Martín J.L., Celemín-Viñuela A., Gómez-Costa D., Zafra-Vallejo M., Agustín-Panadero R., Gómez-Polo M. (2023). Accuracy between 2D Photography and Dual-Structured Light 3D Facial Scanner for Facial Anthropometry: A Clinical Study. J. Clin. Med..

[25] Piedra-Cascón W., Meyer M.J., Methani M.M., Revilla-León M. (2020). Accuracy (Trueness and Precision) of a Dual-Structured Light Facial Scanner and Interexaminer Reliability. J. Prosthet. Dent..

[26] Nguyen M.T., Vu T.T., Nguyen Q.N. (2021). Advanced Digital 3D Technology in the Combined Surgery-First Orthognathic and Clear Aligner Orthodontic Therapy for Dentofacial Deformity Treatment. Processes.

[27] Pandey R., Kamble R., Kanani H. (2024). Revolutionizing Smiles: Advancing Orthodontics Through Digital Innovation. Cureus.

[28] Revilla-León M., Gómez-Polo M., Sailer I., Kois J.C., Rokhshad R. (2024). An Overview of Artificial Intelligence Based Applications for Assisting Digital Data Acquisition and Implant Planning Procedures. J. Esthet. Restor. Dent..

[29] Hung K.F., Yeung A.W.K., Bornstein M.M., Schwendicke F. (2023). Personalized Dental Medicine, Artificial Intelligence, and Their Relevance for Dentomaxillofacial Imaging. Dentomaxillofacial Radiol..

[30] Kois J.C., Zeitler J.M., Barmak A.B., Yilmaz B., Gómez-Polo M., Revilla-León M. (2023). Discrepancies in the Occlusal Devices Designed by an Experienced Dental Laboratory Technician and by 2 Artificial Intelligence-Based Automatic Programs. J. Prosthet. Dent..

[31] Saghiri M.A., Vakhnovetsky J., Samadi E., Amanabi M., Morgano S.M. (2023). C.E. Credit. Innovating Dental Education with Artificial Intelligence. J. Calif. Dent. Assoc..

[32] Cho R.-Y., Byun S.-H., Yi S.-M., Ahn H.-J., Nam Y.-S., Park I.-Y., On S.-W., Kim J.-C., Yang B.-E. (2023). Comparative Analysis of Three Facial Scanners for Creating Digital Twins by Focusing on the Difference in Scanning Method. Bioengineering.

[33] Lee J.-H., Lee H.-L., Park I.-Y., On S.-W., Byun S.-H., Yang B.-E. (2023). Effectiveness of Creating Digital Twins with Different Digital Dentition Models and Cone-Beam Computed Tomography. Sci. Rep..

[34] Saghiri M.A., Vakhnovetsky J., Saghiri A.M. (2023). The Future of Digital Twins in Precision Dentistry. J. Oral Biol. Craniofacial Res..

[35] Doshi K., Sathe S., Dubey S., Bhoyar A., Dhamande M., Jaiswal T. (2024). A Comprehensive Review on Virtual Articulators. Cureus.

[36] Jain A.R., Verma A.C. (2016). Articulators through the Years Revisited: From 1971–1990. Int. J. Pharm Bio. Sci..

[37] Anes L., Cardoso J.A., Azevedo L., Oliveira K., Maligno F. (2024). Mounting Digital Casts on a Virtual Articulator by Using Two-Dimensional Facial Photographs with a Facebow: A Technique. J. Prosthet. Dent..

[38] Maheshwari K., Gupta A.K., Tiwari B. (2024). A Newly Proposed Classification for Articulators-Integrating Virtual with Conventional. J. Indian Prosthodont. Soc..

[39] Goldstein G., Goodacre C. (2023). Selecting a Virtual Articulator: An Analysis of the Factors Available with Mechanical Articulators and Their Potential Need for Inclusion with Virtual Articulators. J. Prosthodont..

[40] Lin H., Pan Y., Wei X., Wang Y., Yu H., Cheng H. (2023). Comparison of the Performance of Various Virtual Articulator Mounting Procedures: A Self-Controlled Clinical Study. Clin. Oral. Invest..

[41] Lepidi L., Galli M., Mastrangelo F., Venezia P., Joda T., Wang H., Li J. (2021). Virtual Articulators and Virtual Mounting Procedures: Where Do We Stand?. J. Prosthodont..

[42] Lepidi L., Chen Z., Ravida A., Lan T., Wang H., Li J. (2019). A Full-Digital Technique to Mount a Maxillary Arch Scan on a Virtual Articulator. J. Prosthodont..

[43] Wang H., Shao L., Sun J., Wang S., Ding Q., Zhang L. (2024). A Fully Digital Workflow to Design Anterior Guidance for an Implant-Supported Single Crown Using a Modified Patient-Specific Motion Technique. J. Prosthet. Dent..

[44] Kim J.-E., Park J.-H., Moon H.-S., Shim J.-S. (2019). Complete Assessment of Occlusal Dynamics and Establishment of a Digital Workflow by Using Target Tracking with a Three-Dimensional Facial Scanner. J. Prosthodont. Res..

[45] Lam W.Y.H., Hsung R.T.C., Choi W.W.S., Luk H.W.K., Cheng L.Y.Y., Pow E.H.N. (2018). A Clinical Technique for Virtual Articulator Mounting with Natural Head Position by Using Calibrated Stereophotogrammetry. J. Prosthet. Dent..

[46] Lam W.Y.H., Hsung R.T.C., Choi W.W.S., Luk H.W.K., Pow E.H.N. (2016). A 2-Part Facebow for CAD-CAM Dentistry. J. Prosthet. Dent..

[47] Solaberrieta E., Mínguez R., Barrenetxea L., Etxaniz O. (2013). Direct Transfer of the Position of Digitized Casts to a Virtual Articulator. J. Prosthet. Dent..

[48] Hong S.-J., Noh K. (2021). Setting the Sagittal Condylar Inclination on a Virtual Articulator by Using a Facial and Intraoral Scan of the Protrusive Interocclusal Position: A Dental Technique. J. Prosthet. Dent..

[49] Lassmann Ł., Nowak Z., Żółtowska A. (2024). Sagittal Condylar Guidance Angle Measurement Methods: A Systematic Review. J. Prosthet. Dent..

[50] Palaskar J.N., Joshi N.P., Hindocha A.D., Gunjal A.P., Balsaraf K.D. (2022). Evaluation of Sagittal Inclination of Occlusal Plane and Horizontal Condylar Guidance Using Various Anterior Reference Points on Arcon and Nonarcon Articulators. Contemp. Clin. Dent..

[51] Das A., Muddugangadhar B.C., Mawani D.P., Mukhopadhyay A. (2021). Comparative Evaluation of Sagittal Condylar Guidance Obtained from a Clinical Method and with Cone Beam Computed Tomography in Dentate Individuals. J. Prosthet. Dent..

[52] Mawani D., Muddugangadhar B., Das A., Mukhopadhyay A. (2019). Comparative Evaluation of Condylar Inclination in Dentulous Subjects as Determined by Two Radiographic Methods: Orthopantomograph and Cone-Beam Computed Tomography—An in Vivo Study. J. Indian. Prosthodont. Soc..

[53] Bapelle M., Dubromez J., Savoldelli C., Tillier Y., Ehrmann E. (2024). Modjaw^®^ Device: Analysis of Mandibular Kinematics Recorded for a Group of Asymptomatic Subjects. CRANIO.

[54] Hong S., Choi Y., Park M., Paek J., Pae A., Kim H., Kwon K., Noh K. (2020). Setting the Sagittal Condylar Inclination on a Virtual Articulator Using Intraoral Scan of Protrusive Interocclusal Position and Cone Beam Computed Tomography. J. Prosthodont..

[55] Ma L., Liu F., Mei J., Chao J., Wang Z., Shen J. (2023). A Comparative Study to Measure the Sagittal Condylar Inclination Using Mechanical Articulator, Virtual Articulator and Jaw Tracking Device. J. Adv. Prosthodont..

[56] Reicheneder C., Gedrange T., Baumert U., Faltermeier A., Proff P. (2009). Variations in the Inclination of the Condylar Path in Children and Adults. Angle Orthod..

[57] Cimić S., Simunković S.K., Kocijan S.S., Matijević J., Dulcić N., Catić A. (2015). Articulator-Related Registration and Analysis of Sagittal Condylar Inclination. Acta Clin. Croat..

[58] Lauren M., McIntyre F. (2013). 4D Clinical Imaging for Dynamic CAD. Int. J. Dent..

[59] Lee Y.-C., Lee C., Shim J.-S., Park J.-M., Shin Y., Kim J.-E., Lee K.-W. (2020). Comparison between Occlusal Errors of Single Posterior Crowns Adjusted Using Patient Specific Motion or Conventional Methods. Appl. Sci..

[60] Valenti M., Schmitz J.H. (2021). A Reverse Digital Workflow by Using an Interim Restoration Scan and Patient-Specific Motion with an Intraoral Scanner. J. Prosthet. Dent..

[61] Revilla-León M., Agustín-Panadero R., Zeitler J.M., Barmak A.B., Yilmaz B., Kois J.C., Pérez-Barquero J.A. (2023). Differences in Maxillomandibular Relationship Recorded at Centric Relation When Using a Conventional Method, Four Intraoral Scanners, and a Jaw Tracking System: A Clinical Study. J. Prosthet. Dent..

[62] Revilla-León M., Pérez-Barquero J.A., Barmak B.A., Agustín-Panadero R., Fernández-Estevan L., Att W. (2021). Facial Scanning Accuracy Depending on the Alignment Algorithm and Digitized Surface Area Location: An in Vitro Study. J. Dent..

[63] Tomar S.S., Rathee M., Diwan K., Senthilvelpalani B. (2024). Unconventional Digital Dentures: Overcoming Software Challenges and Integrating Digital Workflows into Conventional Techniques. J. Prosthet. Dent..

[64] Watanabe H., Fellows C., An H. (2022). Digital Technologies for Restorative Dentistry. Dent. Clin. N. Am..

[65] Koralakunte P.R. (2014). The Role of Virtual Articulator in Prosthetic and Restorative Dentistry. J. Clin. Diagn. Res. JCDR.

[66] Hegedus T., Kreuter P., Kismarczi-Antalffy A.A., Demeter T., Banyai D., Vegh A., Geczi Z., Hermann P., Payer M., Zsembery A. (2022). User Experience and Sustainability of 3D Printing in Dentistry. Int. J. Environ. Res. Public Health.

[67] Khanna R., Konyukhov Y., Maslennikov N., Kolesnikov E., Burmistrov I. (2023). An Overview of Dental Solid Waste Management and Associated Environmental Impacts: A Materials Perspective. Sustainability.

[68] Naderi R.K., Patel T.J., Thompson M.A. (2024). A Comparison Study: The Use of Digital and Conventional Impression Techniques in Dental Hygiene Education. J. Dent. Educ..

[69] Mahmood M., Ijaz M. (2024). Advances in CADCAM Technology for Chairside Restorative Dentistry- A Workflow Analysis. J. Dent. Care.

[70] Guo Y., Juang J., Durham E., Fayyad R., Hackley D. (2024). Assessing the Travel Carbon Footprint of Faculty, Students, and Staff at a U.S. Dental School. J. Calif. Dent. Assoc..

[71] Hsu L.-P., Huang Y.-K., Chang Y.-C. (2022). The Implementation of Artificial Intelligence in Dentistry Could Enhance Environmental Sustainability. J. Dent. Sci..

